# Supplementary material for: Duchenne muscular dystrophy in Italy: A systematic review of epidemiology, quality of life, treatment adherence, and economic impact
Source: PLoS One. 2023 Jun 27;18(6):e0287774. doi: 10.1371/journal.pone.0287774 (PMC10298760; doi:10.1371/journal.pone.0287774)
Supplement: S2 Appendix — (DOCX) [file pone.0287774.s002.docx]

**S2 Appendix. Literature search strategies**

| **PubMed (original search: 25/03/2021; updated search: 16/03/2022; updated search: 31/01/2023)** | | |
| --- | --- | --- |
| Search | Query | Results |
| #1 | duchenne | 13,429 |
| #2 | duchenne muscular dystrophy | 12,722 |
| #3 | DMD | 8,980 |
| #4 | (severe dystrophinopathy AND duchenne) | 131 |
| #5 | becker muscular dystrophy | 13,098 |
| #6 | BMD AND becker | 1,076 |
| #7 | becker dystrophinopathy | 533 |
| #8 | (distrofia muscolare duchenne) OR (distrofinopatia duchenne) OR (distrofia muscolare becker) OR (distrofinopatia becker) | 54 |
| #9 | #1 OR #2 OR #3 OR #4 OR #5 OR #6 OR #7 OR #8 | 16,629 |
| #10 | adherence OR adherent OR compliance OR compliant OR “Medication Possession Ratio” OR “proportion of days covered” OR aderenza OR aderente OR aderenti | 429,843 |
| #11 | epidemiology[Title/Abstract] OR prevalence[Title/Abstract] OR incidence[Title/Abstract] OR mortality[Title/Abstract] OR morbidity[Title/Abstract] OR epidemiologia[Title/Abstract] OR prevalenza[Title/Abstract] OR prevalente[Title/Abstract] OR prevalenti[Title/Abstract] OR incidenza[Title/Abstract] OR incidente[Title/Abstract] OR incidenti[Title/Abstract] OR mortalità[Title/Abstract] OR morbidità[Title/Abstract] OR morbilità[Title/Abstract] | 2,303,240 |
| #12 | cost-effective OR cost-effectiveness OR cost effective OR cost effectiveness OR cost-utility OR cost utility OR cost-benefit OR cost benefit OR cost-consequence OR cost consequence OR cost-analysis OR cost analysis OR cost-analyses OR cost analyses OR economic analysis OR economic analyses OR economic evaluation OR economic evaluations OR economic study OR economic studies OR cost evaluation OR incremental cost-effectiveness ratio OR ICER OR QALY OR QALYs OR quality-adjusted life-years OR quality adjusted life years OR quality-adjusted life-year OR quality adjusted life year OR DALY OR DALYs OR disability-adjusted life-years OR disability adjusted life years OR disability-adjusted life-year OR disability adjusted life year OR direct cost OR direct costs OR indirect cost OR indirect costs OR cost ratio OR cost ratios OR economic impact OR economic burden OR economic benefit OR economic comparison OR economic cost OR economic costs OR economic outcome OR economic outcomes OR economic model OR economic effect OR cost saving OR cost-saving OR cost savings OR willingness pay OR willingness-to-pay OR WTP OR economics[MeSH Terms] OR value of life[MeSH Terms] OR "costs and cost analysis"[MeSH Terms] OR economics, hospital[MeSH Terms] OR economics, medical[MeSH Terms] OR resource allocation[MeSH Terms] OR economics, nursing[MeSH Terms] OR "economics, pharmaceutical"[MeSH Terms] OR "fees and charges"[MeSH Terms] OR budgets[MeSH Terms] OR budget*[Title/Abstract] OR cost*[Title/Abstract] OR (economic*[Title/Abstract] OR pharmaco economic*[Title/Abstract] OR pharmaco-economic*[Title/Abstract] OR pharmacoeconomic*[Title/Abstract]) OR (price*[Title/Abstract] OR pricing*[Title/Abstract]) OR (financ*[Title/Abstract] OR fee[Title/Abstract] OR fees[Title/Abstract] OR expenditure*[Title/Abstract] OR saving*[Title/Abstract]) OR (value[Title/Abstract] AND (money[Title/Abstract] OR monetary[Title/Abstract])) OR resourc* allocat*[Title/Abstract] OR (fund[Title/Abstract] OR funds[Title/Abstract] OR funding*[Title/Abstract] OR funded[Title/Abstract]) OR (ration[Title/Abstract] OR rations[Title/Abstract] OR rationing*[Title/Abstract] OR rationed[Title/Abstract]) OR "economics"[MeSH Subheading] | 1,701,129 |
| #13 | economico OR economici OR economia OR costo OR costi OR costo-efficacia OR costo efficacia OR costi efficacia OR costi-efficacia OR costo-utilità OR costo utilità OR costi-utilità OR costi utilità OR costo-beneficio OR costo beneficio OR costi-benefici OR costi benefici OR costo-opportunità OR costo opportunità OR costi-opportunità OR costi opportunità OR costo incrementale OR costi incrementali OR rapporto costo-efficacia incrementale OR anni di vita aggiustati per la qualità OR anni di vita aggiustati per disabilità OR allocazione delle risorse OR farmaco-economia OR farmacoeconomia OR farmaco-economico OR farmacoeconomico OR prezzo OR prezzi OR spesa OR spese OR risparmio OR risparmi OR valore monetario OR finanziamento OR finanziamenti OR disponibilità pagare | 10,100 |
| #14 | quality of life OR QoL OR value of life OR quality-adjusted life-years OR quality adjusted life years OR quality-adjusted life-year OR quality adjusted life year OR QALY OR QALYs OR disability-adjusted life-years OR disability adjusted life years OR disability-adjusted life-year OR disability adjusted life year OR DALY OR DALYs OR sf36[Title/Abstract] OR "sf 36"[Title/Abstract] OR "short form 36"[Title/Abstract] OR "shortform 36"[Title/Abstract] OR "sf thirtysix"[Title/Abstract] OR "sf thirty six"[Title/Abstract] OR "shortform thirtysix"[Title/Abstract] OR "shortform thirty six"[Title/Abstract] OR "short form thirty six"[Title/Abstract] OR "short form thirtysix"[Title/Abstract] OR "short form thirty six"[Title/Abstract] OR sf6[Title/Abstract] OR "sf 6"[Title/Abstract] OR "SF 6D"[Title/Abstract] OR "short form 6"[Title/Abstract] OR "shortform 6"[Title/Abstract] OR "sf six"[Title/Abstract] OR sfsix[Title/Abstract] OR "shortform six"[Title/Abstract] OR "short form six"[Title/Abstract] OR sf12[Title/Abstract] OR "sf 12"[Title/Abstract] OR "short form 12"[Title/Abstract] OR "shortform 12"[Title/Abstract] OR "sf twelve"[Title/Abstract] OR sftwelve[Title/Abstract] OR "shortform twelve"[Title/Abstract] OR "short form twelve"[Title/Abstract] OR sf16[Title/Abstract] OR "sf 16"[Title/Abstract] OR "short form 16"[Title/Abstract] OR "shortform 16"[Title/Abstract] OR "sf sixteen"[Title/Abstract] OR sfsixteen[Title/Abstract] OR "shortform sixteen"[Title/Abstract] OR "short form sixteen"[Title/Abstract] OR sf20[Title/Abstract] OR "sf 20"[Title/Abstract] OR "short form 20"[Title/Abstract] OR "shortform 20"[Title/Abstract] OR "sf twenty"[Title/Abstract] OR sftwenty[Title/Abstract] OR "shortform twenty"[Title/Abstract] OR "short form twenty"[Title/Abstract] OR euroqol[Title/Abstract] OR "euro qol"[Title/Abstract] OR "eq5d"[Title/Abstract] OR "eq 5d"[Title/Abstract] OR hql[Title/Abstract] OR hqol[Title/Abstract] OR "h qol"[Title/Abstract] OR hrqol[Title/Abstract] OR "hr qol"[Title/Abstract] OR "hye"[Title/Abstract] OR "hyes"[Title/Abstract] OR health* year* equivalent*[Title/Abstract] OR "Health Utilities Index"[Title/Abstract] OR hui[Title/Abstract] OR hui1[Title/Abstract] OR hui2[Title/Abstract] OR hui3[Title/Abstract] OR disutil*[Title/Abstract] OR "quality of well being"[Title/Abstract] OR "quality of wellbeing"[Title/Abstract] OR qwb[Title/Abstract] OR "willingness to pay"[Title/Abstract] OR "willingness-to-pay"[Title/Abstract] OR WTP[Title/Abstract] OR (index[Title/Abstract] AND well being[Title/Abstract]) OR (quality[Title/Abstract] AND well being[Title/Abstract]) OR health status indicator*[Title/Abstract] OR "health related quality of life"[Title/Abstract] OR "health-related quality of life"[Title/Abstract] OR HRQoL[Title/Abstract] OR HR-QoL[Title/Abstract] OR (patient*[Title/Abstract] AND (value[Title/Abstract] OR values[Title/Abstract] OR preference[Title/Abstract] OR preferences[Title/Abstract] OR satisfaction[Title/Abstract] OR acceptance[Title/Abstract])) | 1,308,633 |
| #15 | qualità della vita OR qualità di vita OR benessere OR ((valore OR valori OR preferenza OR preferenze OR desiderio OR desideri OR bisogno OR bisogni OR necessità OR obiettivo OR obiettivi OR convinzione OR convinzioni OR soddisfazione OR accettazione OR consenso) AND (paziente OR pazienti OR malato OR malata OR malati OR cittadino OR cittadina OR cittadini OR persona OR persone OR soggetto OR soggetti OR individuo OR individui OR gruppo OR gruppi OR neonato OR neonata OR neonati OR bambino OR bambina OR bambini OR adolescente OR adolescenti OR adulto OR adulta OR adulti OR anziano OR anziana OR anziani)) OR stato di salute | 1,245 |
| #16 | Italia OR Italy OR Italian OR italiano OR italiana OR Lombardia OR Lombardy OR Lazio OR Veneto OR Emilia-Romagna OR "Emilia Romagna" OR Piemonte OR Piedmont OR Toscana OR Tuscany OR Campania OR Sicilia OR "Regione Siciliana" OR Sicily OR Puglia OR Apulia OR Liguria OR "Marche Region" OR "Friuli-Venezia Giulia" OR "Friuli Venezia Giulia" OR Sardegna OR Sardinia OR Abruzzo OR Calabria OR "Trentino-Alto Adige" OR "Trentino Alto Adige" OR Trentino-Südtirol OR "Trentino South-Tyrol" OR Bolzano OR Umbria OR Trento OR Basilicata OR Molise OR "Valle d'Aosta" OR "Aosta Valley" OR "Vallée d'Aoste" OR Bergamo OR Brescia OR Como OR Cremona OR Lecco OR Lodi OR Mantova OR Milano OR Milan OR "Monza e Brianza" OR Pavia OR Sondrio OR Varese OR Frosinone OR Latina OR Rieti OR Roma OR Rome OR Viterbo OR Avellino OR Benevento OR Caserta OR Napoli OR Naples OR Salerno OR Agrigento OR Caltanissetta OR Catania OR Enna OR Messina OR Palermo OR Ragusa OR Siracusa OR Trapani OR Belluno OR Padova OR Rovigo OR Treviso OR Venezia OR Venice OR Verona OR Vicenza OR Bologna OR Ferrara OR Forlì-Cesena OR Modena OR Parma OR Piacenza OR Ravenna OR "Reggio Emilia" OR Rimini OR Alessandria OR Asti OR Biella OR Cuneo OR Novara OR Torino OR Turin OR Verbano-Cusio-Ossola OR Vercelli OR Bari OR Barletta-Andria-Trani OR Brindisi OR Foggia OR Lecce OR Taranto OR Arezzo OR Firenze OR Florence OR Grosseto OR Livorno OR Lucca OR "Massa e Carrara" OR Pisa OR Pistoia OR Prato OR Siena OR Catanzaro OR Cosenza OR Crotone OR "Reggio Calabria" OR "Vibo Valentia" OR Cagliari OR Nuoro OR Oristano OR Sassari OR "Sud Sardegna" OR Genova OR Genoa OR Imperia OR "La Spezia" OR Savona OR Ancona OR "Ascoli Piceno" OR Fermo OR Macerata OR "Pesaro e Urbino" OR Chieti OR "L'Aquila" OR Pescara OR Teramo OR Trieste OR Perugia OR Terni OR Matera OR Potenza OR Campobasso OR Isernia OR Aosta | 1,312,227 |
| #17 | Adherence  #9 AND #10 AND #16  Filters: English, Italian, from January 2010 – March 2021  **Update 16/03/2022**: Filters: English, Italian, from March 2021 to March 2022  **Update 31/01/2023**: Filters: English, Italian, from March 2022 to 31 January 2023 | 19  1  3 |
| #18 | Epidemiology  #9 AND #11 AND #16  Filters: English, Italian, from January 2010 – March 2021  **Update 16/03/2022**: Filters: English, Italian, from March 2021 to March 2022  **Update 31/01/2023**: Filters: English, Italian, from March 2022 to 31 January 2023 | 46  8  7 |
| #19 | Economic studies  #9 AND (#12 OR #13) AND #16  Filters: English, Italian, from January 2010 – March 2021  **Update 16/03/2022**: Filters: English, Italian, from March 2021 to March 2022  **Update 31/01/2023**: Filters: English, Italian, from March 2022 to 31 January 2023 | 35  7  5 |
| #20 | Quality of life  #9 AND (#14 OR #15) AND #16  Filters: English, Italian, from January 2010 – March 2021  **Update 16/03/2022**: Filters: English, Italian, from March 2021 to March 2022  **Update 31/01/2023**: Filters: English, Italian, from March 2022 to 31 January 2023 | 72  22  16 |
| #21 | All results  #17 OR #18 OR #19 OR #20  Filters: English, Italian, from January 2010 – March 2021  **Update 16/03/2022**: Filters: English, Italian, from March 2021 to March 2022  **Update 31/01/2023**: Filters: English, Italian, from March 2022 to 31 January 2023 | 149  27  23 |

| **Embase (original search: 25/03/2021; updated search: 16/03/2022; updated search: 31/01/2023)** | | |
| --- | --- | --- |
| Search | Query | Results |
| #1 | duchenne | 21,516 |
| #2 | ‘duchenne muscular dystrophy’ | 19,522 |
| #3 | DMD | 20,123 |
| #4 | (severe AND dystrophinopathy AND duchenne) | 115 |
| #5 | becker AND muscular AND dystrophy | 4,161 |
| #6 | BMD AND becker | 1,496 |
| #7 | becker AND dystrophinopathy | 394 |
| #8 | (distrofia AND muscolare AND duchenne) OR (distrofinopatia AND duchenne) OR (distrofia AND muscolare AND becker) OR (distrofinopatia AND becker) | 96 |
| #9 | #1 OR #2 OR #3 OR #4 OR #5 OR #6 OR #7 OR #8 | 32,907 |
| #10 | ('patient compliance'/exp OR 'patient compliance' OR adherence OR adherent OR compliance OR compliant OR 'medication possession ratio' OR 'proportion of days covered' OR aderenza OR aderente OR aderenti) | 558,128 |
| #11 | (epidemiology:ti,ab,kw OR prevalence:ti,ab,kw OR incidence:ti,ab,kw OR mortality:ti,ab,kw OR morbidity:ti,ab,kw OR epidemiologia:ti,ab,kw OR prevalenza:ti,ab,kw OR prevalente:ti,ab,kw OR prevalenti:ti,ab,kw OR incidenza:ti,ab,kw OR incidente:ti,ab,kw OR incidenti:ti,ab,kw OR mortalità:ti,ab,kw OR morbidità:ti,ab,kw OR morbilità:ti,ab,kw) | 3,288,787 |
| #12 | ('cost-effective' OR 'cost-effectiveness' OR 'cost effective' OR 'cost effectiveness' OR 'cost-utility' OR 'cost utility' OR 'cost-benefit' OR 'cost benefit' OR 'cost-consequence' OR 'cost consequence' OR 'cost-analysis' OR 'cost analysis' OR 'cost-analyses' OR 'cost analyses' OR 'economic analysis' OR 'economic analyses' OR 'economic evaluation' OR 'economic evaluations' OR 'economic study' OR 'economic studies' OR 'cost evaluation' OR 'incremental cost-effectiveness ratio' OR icer OR qaly OR qalys OR 'quality-adjusted life-years' OR 'quality adjusted life years' OR 'quality-adjusted life-year' OR 'quality adjusted life year' OR daly OR dalys OR 'disability-adjusted life-years' OR 'disability adjusted life years' OR 'disability-adjusted life-year' OR 'disability adjusted life year' OR 'direct cost' OR 'direct costs' OR 'indirect cost' OR 'indirect costs' OR 'cost ratio' OR 'cost ratios' OR 'economic impact' OR 'economic burden' OR 'economic benefit' OR 'economic comparison' OR 'economic cost' OR 'economic costs' OR 'economic outcome' OR 'economic outcomes' OR 'economic model' OR 'economic effect' OR 'cost saving' OR 'cost-saving' OR 'cost savings' OR 'willingness to pay' OR 'willingness-to-pay' OR wtp OR 'economics'/exp OR 'value of life'/exp OR 'economic aspect'/exp OR budget*:ti,ab OR cost*:ti,ab OR economic*:ti,ab OR 'pharmaco economic*':ti,ab OR pharmacoeconomic*:ti,ab OR price*:ti,ab OR pricing*:ti,ab OR financ*:ti,ab OR fee:ti,ab OR fees:ti,ab OR expenditure*:ti,ab OR saving*:ti,ab OR (value:ti,ab AND (money:ti,ab OR monetary:ti,ab)) OR 'resourc* allocat*':ti,ab OR fund:ti,ab OR funds:ti,ab OR funding*:ti,ab OR funded:ti,ab OR ration:ti,ab OR rations:ti,ab OR rationing*:ti,ab OR rationed:ti,ab) | 2,713,457 |
| #13 | (economico OR economici OR economia OR costo OR costi OR 'costo-efficacia' OR 'costo efficacia' OR 'costi efficacia' OR 'costi-efficacia' OR 'costo-utilità' OR 'costo utilità' OR 'costi-utilità' OR 'costi utilità' OR 'costo-beneficio' OR 'costo beneficio' OR 'costi-benefici' OR 'costi benefici' OR 'costo-opportunità' OR 'costo opportunità' OR 'costi-opportunità' OR 'costi opportunità' OR 'costo incrementale' OR 'costi incrementali' OR 'rapporto costo-efficacia incrementale' OR 'anni di vita aggiustati per la qualità' OR 'anni di vita aggiustati per disabilità' OR 'allocazione delle risorse' OR 'farmaco-economia' OR 'farmacoeconomia' OR 'farmaco-economico' OR farmacoeconomico OR prezzo OR prezzi OR spesa OR spese OR risparmio OR risparmi OR 'valore monetario' OR finanziamento OR finanziamenti OR 'disponibilità a pagare') | 9,000 |
| #14 | ('quality of life'/exp OR 'quality of life' OR qol OR 'value of life'/exp OR 'quality-adjusted life-years' OR 'quality adjusted life years' OR 'quality-adjusted life-year' OR 'quality adjusted life year' OR qaly OR qalys OR 'disability-adjusted life-years' OR 'disability adjusted life years' OR 'disability-adjusted life-year' OR 'disability adjusted life year' OR daly OR dalys OR sf36:ti,ab OR 'sf 36':ti,ab OR 'short form 36':ti,ab OR 'shortform 36':ti,ab OR 'sf thirtysix':ti,ab OR 'sf thirty six':ti,ab OR 'shortform thirtysix':ti,ab OR 'shortform thirty six':ti,ab OR 'short form thirtysix':ti,ab OR 'short form thirty six':ti,ab OR sf6:ti,ab OR 'sf 6':ti,ab OR 'sf 6d':ti,ab OR 'short form 6':ti,ab OR 'shortform 6':ti,ab OR 'sf six':ti,ab OR sfsix:ti,ab OR 'shortform six':ti,ab OR 'short form six':ti,ab OR sf12:ti,ab OR 'sf 12':ti,ab OR 'short form 12':ti,ab OR 'shortform 12':ti,ab OR 'sf twelve':ti,ab OR sftwelve:ti,ab OR 'shortform twelve':ti,ab OR 'short form twelve':ti,ab OR sf16:ti,ab OR 'sf 16':ti,ab OR 'short form 16':ti,ab OR 'shortform 16':ti,ab OR 'sf sixteen':ti,ab OR sfsixteen:ti,ab OR 'shortform sixteen':ti,ab OR 'short form sixteen':ti,ab OR sf20:ti,ab OR 'sf 20':ti,ab OR 'short form 20':ti,ab OR 'shortform 20':ti,ab OR 'sf twenty':ti,ab OR sftwenty:ti,ab OR 'shortform twenty':ti,ab OR 'short form twenty':ti,ab OR euroqol:ti,ab OR 'euro qol':ti,ab OR 'eq5d':ti,ab OR 'eq 5d':ti,ab OR hql:ti,ab OR hqol:ti,ab OR 'h qol':ti,ab OR 'hr qol':ti,ab OR 'hye':ti,ab OR 'hyes':ti,ab OR 'health* year* equivalent*':ti,ab OR 'health utilities index':ti,ab OR hui:ti,ab OR hui1:ti,ab OR hui2:ti,ab OR hui3:ti,ab OR disutil*:ti,ab OR 'quality of well being':ti,ab OR 'quality of wellbeing':ti,ab OR qwb:ti,ab OR 'willingness to pay':ti,ab OR 'willingness-to-pay':ti,ab OR wtp:ti,ab OR (index:ti,ab AND 'well being':ti,ab) OR (quality:ti,ab AND 'well being':ti,ab) OR 'health status indicator*':ti,ab OR 'health related quality of life':ti,ab OR 'health-related quality of life':ti,ab OR hrqol:ti,ab OR 'hr-qol':ti,ab OR (patient*:ti,ab AND (value:ti,ab OR values:ti,ab OR preference:ti,ab OR preferences:ti,ab OR satisfaction:ti,ab OR acceptance:ti,ab))) | 2,377,789 |
| #15 | ('qualità della vita' OR 'qualità di vita' OR benessere OR ((valore OR valori OR preferenza OR preferenze OR desiderio OR desideri OR bisogno OR bisogni OR necessità OR obiettivo OR obiettivi OR convinzione OR convinzioni OR soddisfazione OR accettazione OR consenso) AND (paziente OR pazienti OR malato OR malata OR malati OR cittadino OR cittadina OR cittadini OR persona OR persone OR soggetto OR soggetti OR individuo OR individui OR gruppo OR gruppi OR neonato OR neonata OR neonati OR bambino OR bambina OR bambini OR adolescente OR adolescenti OR adulto OR adulta OR adulti OR anziano OR anziana OR anziani)) OR 'stato di salute') | 1,978 |
| #16 | (italia OR italy OR italian OR italiano OR italiana OR lombardia OR lombardy OR lazio OR veneto OR 'emilia-romagna' OR 'emilia romagna' OR piemonte OR piedmont OR toscana OR tuscany OR campania OR sicilia OR 'regione siciliana' OR sicily OR puglia OR apulia OR liguria OR 'marche region' OR 'friuli-venezia giulia' OR 'friuli venezia giulia' OR sardegna OR sardinia OR abruzzo OR calabria OR 'trentino-alto adige' OR 'trentino alto adige' OR 'trentino-südtirol' OR 'trentino south-tyrol' OR bolzano OR umbria OR trento OR basilicata OR molise OR 'valle aosta' OR 'aosta valley' OR 'vallée aoste' OR bergamo OR brescia OR como OR cremona OR lecco OR lodi OR mantova OR milano OR milan OR 'monza e brianza' OR pavia OR sondrio OR varese OR frosinone OR latina OR rieti OR roma OR rome OR viterbo OR avellino OR benevento OR caserta OR napoli OR naples OR salerno OR agrigento OR caltanissetta OR catania OR enna OR messina OR palermo OR ragusa OR siracusa OR trapani OR belluno OR padova OR rovigo OR treviso OR venezia OR venice OR verona OR vicenza OR bologna OR ferrara OR 'forlì-cesena' OR modena OR parma OR piacenza OR ravenna OR 'reggio emilia' OR rimini OR alessandria OR asti OR biella OR cuneo OR novara OR torino OR turin OR 'verbano-cusio-ossola' OR vercelli OR bari OR 'barletta-andria-trani' OR brindisi OR foggia OR lecce OR taranto OR arezzo OR firenze OR florence OR grosseto OR livorno OR lucca OR 'massa e carrara' OR pisa OR pistoia OR prato OR siena OR catanzaro OR cosenza OR crotone OR 'reggio calabria' OR 'vibo valentia' OR cagliari OR nuoro OR oristano OR sassari OR 'sud sardegna' OR genova OR genoa OR imperia OR 'la spezia' OR savona OR ancona OR 'ascoli piceno' OR fermo OR macerata OR 'pesaro e urbino' OR chieti OR 'aquila' OR pescara OR teramo OR trieste OR perugia OR terni OR matera OR potenza OR campobasso OR isernia OR aosta) | 2,207,052 |
| #17 | Adherence  (#9 AND #10 AND #16) AND ([english]/lim OR [italian]/lim) AND [2010-2021]/py  **Update 16/03/2022**: #17 AND [01-03-2021]/sd NOT [16-03-2022]/sd  **Update 31/01/2023**: #17 AND [16-03-2022]/sd NOT [01-02-2023]/sd | 42  3  7 |
| #18 | Epidemiology  (#9 AND #11 AND #16) AND ([english]/lim OR [italian]/lim) AND [2010-2021]/py  **Update 16/03/2022**: #18 AND [01-03-2021]/sd NOT [16-03-2022]/sd  **Update 31/01/2023**: #18 AND [16-03-2022]/sd NOT [01-02-2023]/sd | 147  11  20 |
| #19 | Economic studies  (#9 AND (#12 OR #13) AND #16) AND ([english]/lim OR [italian]/lim) AND [2010-2021]/py  **Update 16/03/2022**: #19 AND [01-03-2021]/sd NOT [16-03-2022]/sd  **Update 31/01/2023**: #19 AND [16-03-2022]/sd NOT [01-02-2023]/sd | 139  13  13 |
| #20 | Quality of life  (#9 AND (#14 OR #15) AND #16) AND ([english]/lim OR [italian]/lim) AND [2010-2021]/py  **Update 16/03/2022**: #20 AND [01-03-2021]/sd NOT [16-03-2022]/sd  **Update 31/01/2023**: #20 AND [16-03-2022]/sd NOT [01-02-2023]/sd | 260  43  48 |
| #21 | All results  (#17 OR #18 OR #19 OR #20) AND ([article]/lim OR [article in press]/lim OR [review]/lim)  **Update 16/03/2022**: #21 AND [01-03-2021]/sd NOT [16-03-2022]/sd  **Update 31/01/2023**: #21 AND [16-03-2022]/sd NOT [01-02-2023]/sd | 230  59  37 |

| **Web of Science (original search: 25/03/2021; updated search: 16/03/2022; updated search: 31/01/2023)** | | |
| --- | --- | --- |
| Search | Query | Results |
| #1 | TS=(duchenne OR (duchenne AND muscular AND dystrophy) OR DMD OR (severe AND dystrophinopathy AND duchenne) OR (becker AND muscular AND dystrophy) OR (BMD AND becker) OR (becker dystrophinopathy) OR ((distrofia AND muscolare AND duchenne) OR (distrofinopatia AND duchenne) OR (distrofia AND muscolare AND becker) OR (distrofinopatia AND becker))) | 21,867 |
| #2 | TS=(“patient compliance” OR adherence OR adherent OR compliance OR compliant OR “medication possession ratio” OR “proportion of days covered” OR aderenza OR aderente OR aderenti) | 403,974 |
| #3 | TS=(epidemiology OR prevalence OR incidence OR mortality OR morbidity OR epidemiologia OR prevalenza OR prevalente OR prevalenti OR incidenza OR incidente OR incidenti OR mortalità OR morbidità OR morbilità) | 2,782,304 |
| #4 | TS=(“cost-effective” OR “cost-effectiveness” OR “cost effective” OR “cost effectiveness” OR “cost-utility” OR “cost utility” OR “cost-benefit” OR “cost benefit” OR “cost-consequence” OR “cost consequence” OR “cost-analysis” OR “cost analysis” OR “cost-analyses” OR “cost analyses” OR “economic analysis” OR “economic analyses” OR “economic evaluation” OR “economic evaluations” OR “economic study” OR “economic studies” OR “cost evaluation” OR “incremental cost-effectiveness ratio” OR icer OR qaly OR qalys OR “quality-adjusted life-years” OR “quality adjusted life years” OR “quality-adjusted life-year” OR “quality adjusted life year” OR daly OR dalys OR “disability-adjusted life-years” OR “disability adjusted life years” OR “disability-adjusted life-year” OR “disability adjusted life year” OR “direct cost” OR “direct costs” OR “indirect cost” OR “indirect costs” OR “cost ratio” OR “cost ratios” OR “economic impact” OR “economic burden” OR “economic benefit” OR “economic comparison” OR “economic cost” OR “economic costs” OR “economic outcome” OR “economic outcomes” OR “economic model” OR “economic effect” OR “cost saving” OR “cost-saving” OR “cost savings” OR “willingness to pay” OR “willingness-to-pay” OR wtp OR “economics” OR “value of life” OR “economic aspect” OR budget* OR cost* OR economic* OR “pharmaco economic*” OR pharmacoeconomic* OR price* OR pricing* OR financ* OR fee OR fees OR expenditure* OR saving* OR (value AND (money OR monetary)) OR “resourc* allocat*” OR fund OR funds OR funding* OR funded OR ration OR rations OR rationing* OR rationed) | 3,696,951 |
| #5 | TS=(economico OR economici OR economia OR costo OR costi OR “costo-efficacia” OR “costo efficacia” OR “costi efficacia” OR “costi-efficacia” OR “costo-utilità” OR “costo utilità” OR “costi-utilità” OR “costi utilità” OR “costo-beneficio” OR “costo beneficio” OR “costi-benefici” OR “costi benefici” OR “costo-opportunità” OR “costo opportunità” OR “costi-opportunità” OR “costi opportunità” OR “costo incrementale” OR “costi incrementali” OR “rapporto costo-efficacia incrementale” OR “anni di vita aggiustati per la qualità” OR “anni di vita aggiustati per disabilità” OR “allocazione delle risorse” OR “farmaco-economia” OR “farmacoeconomia” OR “farmaco-economico” OR farmacoeconomico OR prezzo OR prezzi OR spesa OR spese OR risparmio OR risparmi OR “valore monetario” OR finanziamento OR finanziamenti OR “disponibilità a pagare”) | 1,188 |
| #6 | TS=(“quality of life” OR qol OR “value of life” OR “quality-adjusted life-years” OR “quality adjusted life years” OR “quality-adjusted life-year” OR “quality adjusted life year” OR qaly OR qalys OR “disability-adjusted life-years” OR “disability adjusted life years” OR “disability-adjusted life-year” OR “disability adjusted life year” OR daly OR dalys OR sf36 OR “sf 36” OR “short form 36” OR “shortform 36” OR “sf thirtysix” OR “sf thirty six” OR “shortform thirtysix” OR “shortform thirty six” OR “short form thirtysix” OR “short form thirty six” OR sf6 OR “sf 6” OR “sf 6d” OR “short form 6” OR “shortform 6” OR “sf six” OR sfsix OR “shortform six” OR “short form six” OR sf12 OR “sf 12” OR “short form 12” OR “shortform 12” OR “sf twelve” OR sftwelve OR “shortform twelve” OR “short form twelve” OR sf16 OR “sf 16” OR “short form 16” OR “shortform 16” OR “sf sixteen” OR sfsixteen OR “shortform sixteen” OR “short form sixteen” OR sf20 OR “sf 20” OR “short form 20” OR “shortform 20” OR “sf twenty” OR sftwenty OR “shortform twenty” OR “short form twenty” OR euroqol OR “euro qol” OR “eq5d” OR “eq 5d” OR hql OR hqol OR “h qol” OR “hr qol” OR “hye” OR “hyes” OR “health* year* equivalent*” OR “health utilities index” OR hui OR hui1 OR hui2 OR hui3 OR disutil* OR “quality of well being” OR “quality of wellbeing” OR qwb OR “willingness to pay” OR “willingness-to-pay” OR wtp OR (index AND “well being”) OR (quality AND “well being”) OR “health status indicator*” OR “health related quality of life” OR “health-related quality of life” OR hrqol OR “hr-qol” OR (patient* AND (value OR values OR preference OR preferences OR satisfaction OR acceptance))) | 1,205,295 |
| #7 | TS=(“qualità della vita” OR “qualità di vita” OR benessere OR ((valore OR valori OR preferenza OR preferenze OR desiderio OR desideri OR bisogno OR bisogni OR necessità OR obiettivo OR obiettivi OR convinzione OR convinzioni OR soddisfazione OR accettazione OR consenso) AND (paziente OR pazienti OR malato OR malata OR malati OR cittadino OR cittadina OR cittadini OR persona OR persone OR soggetto OR soggetti OR individuo OR individui OR gruppo OR gruppi OR neonato OR neonata OR neonati OR bambino OR bambina OR bambini OR adolescente OR adolescenti OR adulto OR adulta OR adulti OR anziano OR anziana OR anziani)) OR “stato di salute”) | 58 |
| #8 | TS=(italia OR italy OR italian OR italiano OR italiana OR lombardia OR lombardy OR lazio OR veneto OR “emilia-romagna” OR “emilia romagna” OR piemonte OR piedmont OR toscana OR tuscany OR campania OR sicilia OR “regione siciliana” OR sicily OR puglia OR apulia OR liguria OR “marche region” OR “friuli-venezia giulia” OR “friuli venezia giulia” OR sardegna OR sardinia OR abruzzo OR calabria OR “trentino-alto adige” OR “trentino alto adige” OR “trentino-südtirol” OR “trentino south-tyrol” OR bolzano OR umbria OR trento OR basilicata OR molise OR “valle aosta” OR “aosta valley” OR “vallée aoste” OR bergamo OR brescia OR como OR cremona OR lecco OR lodi OR mantova OR milano OR milan OR “monza e brianza” OR pavia OR sondrio OR varese OR frosinone OR latina OR rieti OR roma OR rome OR viterbo OR avellino OR benevento OR caserta OR napoli OR naples OR salerno OR agrigento OR caltanissetta OR catania OR enna OR messina OR palermo OR ragusa OR siracusa OR trapani OR belluno OR padova OR rovigo OR treviso OR venezia OR venice OR verona OR vicenza OR bologna OR ferrara OR “forlì-cesena” OR modena OR parma OR piacenza OR ravenna OR “reggio emilia” OR rimini OR alessandria OR asti OR biella OR cuneo OR novara OR torino OR turin OR “verbano-cusio-ossola” OR vercelli OR bari OR “barletta-andria-trani” OR brindisi OR foggia OR lecce OR taranto OR arezzo OR firenze OR florence OR grosseto OR livorno OR lucca OR “massa e carrara” OR pisa OR pistoia OR prato OR siena OR catanzaro OR cosenza OR crotone OR “reggio calabria” OR “vibo valentia” OR cagliari OR nuoro OR oristano OR sassari OR “sud sardegna” OR genova OR genoa OR imperia OR “la spezia” OR savona OR ancona OR “ascoli piceno” OR fermo OR macerata OR “pesaro e urbino” OR chieti OR “aquila” OR pescara OR teramo OR trieste OR perugia OR terni OR matera OR potenza OR campobasso OR isernia OR aosta) | 452,324 |
| #9 | Adherence  (#1 AND #2 AND #8) AND LANGUAGE: (English OR Italian) AND DOCUMENT TYPES: (Article OR Review)  Indexes=SCI-EXPANDED, SSCI, A&HCI, CPCI-S, CPCI-SSH, ESCI Timespan=2010-2021  **Update 16/03/2022**: Timespan=from March 2021 to March 2022  **Update 31/01/2023**: Timespan=from March 2022 to January 2023 | 1  0  0 |
| #10 | Epidemiology  (#1 AND #3 AND #8) AND LANGUAGE: (English OR Italian) AND DOCUMENT TYPES: (Article OR Review)  Indexes=SCI-EXPANDED, SSCI, A&HCI, CPCI-S, CPCI-SSH, ESCI Timespan=2010-2021  **Update 16/03/2022**: Timespan=from March 2021 to March 2022  **Update 31/01/2023**: Timespan=from March 2022 to January 2023 | 6  3  1 |
| #11 | Economic studies  (#1 AND (#4 OR #5) AND #8) AND LANGUAGE: (English OR Italian) AND DOCUMENT TYPES: (Article OR Review)  Indexes=SCI-EXPANDED, SSCI, A&HCI, CPCI-S, CPCI-SSH, ESCI Timespan=2010-2021  **Update 16/03/2022**: Timespan=from March 2021 to March 2022  **Update 31/01/2023**: Timespan=from March 2022 to January 2023 | 7  2  0 |
| #12 | Quality of life  (#1 AND (#6 OR #7) AND #8) AND LANGUAGE: (English OR Italian) AND DOCUMENT TYPES: (Article OR Review)  Indexes=SCI-EXPANDED, SSCI, A&HCI, CPCI-S, CPCI-SSH, ESCI Timespan=2010-2021  **Update 16/03/2022**: Timespan=from March 2021 to March 2022  **Update 31/01/2023**: Timespan=from March 2022 to January 2023 | 14  3  1 |
| #13 | All results  #9 OR #10 OR #11 OR #12  **Update 16/03/2022**: Timespan=from March 2021 to March 2022  **Update 31/01/2023**: Timespan=from March 2022 to January 2023 | 18  5  2 |
