# Supplementary material for: Duchenne muscular dystrophy in Italy: A systematic review of epidemiology, quality of life, treatment adherence, and economic impact
Source: PLoS One. 2023 Jun 27;18(6):e0287774. doi: 10.1371/journal.pone.0287774 (PMC10298760; doi:10.1371/journal.pone.0287774)
Supplement: S3 Appendix — (DOCX) [file pone.0287774.s003.docx]

**S3 Appendix. Excluded studies**

|  | Reference | Reason for exclusion |
| --- | --- | --- |
| 1 | Ambrosini, A., et al. (2019). Be an ambassador for change that you would like to see": a call to action to all stakeholders for co-creation in healthcare and medical research to improve quality of life of people with a neuromuscular disease. Orphanet J Rare Dis 14. | The article describes a workshop and does not provide data of interest for this review. |
| 2 | Arikian, A., et al. (2010). Targeting parents for the treatment of pediatric obesity in boys with Duchenne muscular dystrophy: A case series. Eating and Weight Disorders 15(3): e161-e165. | The study is conducted in the United States and therefore does not report Italian data. |
| 3 | Bell, J. M., et al. (2017). Interventions to prevent and treat corticosteroid-induced osteoporosis and prevent osteoporotic fractures in Duchenne muscular dystrophy. Cochrane Database Syst Rev 1(1): Cd010899. | Systematic review not reporting QoL data. |
| 4 | Brogna, C., et al. (2020). Respiratory function and therapeutic expectations in DMD: families experience and perspective. Acta Myol 39(3): 121-129. | The article does not provide QoL measures. |
| 5 | Campbell, C., et al. (2021). Health related quality of life in young, steroid-naïve boys with Duchenne muscular dystrophy. Neuromuscul Disord 31(11): 1161-1168. | Multicenter study that also includes Italian patients; the results are not described separately for each country. |
| 6 | Catalano, A., et al. (2016). Effects of teriparatide on bone mineral density and quality of life in Duchenne muscular dystrophy related osteoporosis: a case report. Osteoporos Int 27(12): 3655-3659. | Study design: case report. |
| 7 | Claeys, K. (2021). Outcome measures in muscle disorders. Journal of the Neurological Sciences 429. | This is a conference abstract, reporting insufficient data. |
| 8 | Fabriani, V., et al. (2014). Cost of Illness Analysis of Duchenne Muscular Dystrophy In Italy. Value Health 17(7): A528. | This is a conference abstract, reporting insufficient data. We briefly described this study in the Discussion. |
| 9 | Fiorentino, G., et al. (2016). Mouthpiece ventilation in Duchenne muscular dystrophy: a rescue strategy for noncompliant patients. J Bras Pneumol 42(6): 453-456. | The study does not report data on adherence. |
| 10 | Garegnani, L., et al. (2021). Antioxidants to prevent respiratory decline in people with Duchenne muscular dystrophy and progressive respiratory decline. Cochrane Database Syst Rev 12(12): Cd013720. | One of the two studies included in this review (DELOS 2015) is multicentre and also includes Italian patients. However, the results for Italy are not described separately. |
| 11 | Landfeldt, E., et al. (2018). Duchenne muscular dystrophy and caregiver burden: a systematic review. Dev Med Child Neurol 60(10): 987-996. | This systematic review includes Italian studies already included in our review, plus a further study which, however, does not report stratified data for Italy. |
| 12 | Landfeldt, E., et al. (2016). Health-related quality of life in patients with Duchenne muscular dystrophy: a multinational, cross-sectional study. Dev Med Child Neurol 58(5): 508-515. | The study does not report stratified data for Italy. |
| 13 | Lionarons, J. M., et al. (2021). Prevalence of Bladder and Bowel Dysfunction in Duchenne Muscular Dystrophy Using the Childhood Bladder and Bowel Dysfunction Questionnaire. Life (Basel) 11(8). | This study reports data from the Netherlands. |
| 14 | Magliano, L., et al. (2014). "I have got something positive out of this situation": psychological benefits of caregiving in relatives of young people with muscular dystrophy. J Neurol 261(1): 188-195. | The article does not provide QoL measures. |
| 15 | Magliano, L. and L. Politano (2016). Family context in muscular dystrophies: psychosocial aspects and social integration. Acta Myol 35(2): 96-99. | Summary article of studies already included. |
| 16 | Magliano, L., et al. (2017). Integrated care of muscular dystrophies in Italy. Part 2. Psychological treatments, social and welfare support, and financial costs. Acta Myol 36(2): 41-45. | Study that does not report costs (monetary values) but only the percentages of specific categories of resources used (e.g. training, psychological support, social services). |
| 17 | Matsumura, T., et al. (2021). Questionnaire survey on the impact of coronavirus disease 2019 on patients with muscular dystrophy. Journal of the Neurological Sciences 429. | Conference proceeding reporting data on Japanese patients. |
| 18 | Politano, L., et al. (2017). Integrated care of muscular dystrophies in Italy. Part 1. Pharmacological treatment and rehabilitative interventions. Acta Myologica 36(1): 19-24. | The article reports data already described in other articles included without adding other data of interest for this review. |
| 19 | Sequeira, A. R., et al. (2021). The economic and health impact of rare diseases: A meta-analysis. Health Policy and Technology 10(1): 32-44. | Only one Italian study included about DMD (Cavazza 2016), that is already included in our review. |
| 20 | Tesei, A., et al. (2020). Mental health and coping strategies in families of children and young adults with muscular dystrophies. J Neurol 267(7): 2054-2069. | The article does not provide QoL measures. |
| 21 | Topaloglu, H. (2013). Epidemiology of muscular dystrophies in the mediterranean area. Acta Myologica 32(3): 138-141. | Narrative review that does not include any further studies than those described in Crisafulli 2020 and Theadom 2014, except the study by Mostacciuolo et al. (1993) which has been included in our review. |
| 22 | Vita, G. L., et al. (2020). Psychosocial impact of sport activity in neuromuscular disorders. Neurol Sci 41(9): 2561-2567. | The study reports only pooled data for patients with various types of muscular dystrophy. |
