# Supplementary material for: Duchenne muscular dystrophy in Italy: A systematic review of epidemiology, quality of life, treatment adherence, and economic impact
Source: PLoS One. 2023 Jun 27;18(6):e0287774. doi: 10.1371/journal.pone.0287774 (PMC10298760; doi:10.1371/journal.pone.0287774)
Supplement: S4 Appendix — (DOCX) [file pone.0287774.s004.docx]

**S4 Appendix. Quality assessment.**

Research question 1 (epidemiology)

The methodological quality of the two included systematic reviews was assessed using the JBI Critical Appraisal Checklist for Systematic Reviews and Research Synthesis [15], that comprises 11 items with four possible answers: yes, no, unclear, not applicable. The review by Crisafulli et al. [3] satisfied 10/11 (91%) of the checklist items. We judged the item #5 of the checklist (“Were the criteria for appraising studies appropriate?”) partly fulfilled, because the authors assessed the quality of reporting of the included studies using a modified STrengthening the Reporting of OBservational studies in Epidemiology (STROBE) checklist, rather than assessing the risk of bias using an appropriate quality assessment/critical appraisal tool.

The review by Theadom et al. [19] met 5/11 (45%) checklist items. Full search strategies were not reported; search terms reported in the methods seem limited. No formal tool was used for the quality assessment of included studies. Studies were classified as having a low, unclear or high risk of bias based on the risk of underestimating or overestimating the prevalence. No information was reported about the data extraction process and if two authors independently performed the critical appraisal. No meta-analyses were performed, nor the reason why they were not attempted (e.g., high heterogeneity of included studies) was specified. Lastly, the risk of publication bias was not assessed.

The methodological quality of the study by Mostacciuolo et al. of 1993 [20] was assessed by the checklist of Hoy et al. [16]. Almost all checklist items (8/9; 89%) were met, while one item was deemed not applicable (the response rate). The only critical point concerns the generalizability of the results of the study which is considered limited, as the included population comes from five hospitals in the Veneto region and therefore the epidemiological estimates cannot be generalized at national level.

Research question 2 (quality of life)

The methodological quality of the included studies was assessed using the CASP Qualitative Studies Checklist tool [17], that comprises 10 items with three possible answers: yes, no, can’t tell. Most of the studies (6/8; 75%) [28, 30, 31, 33-35] fulfilled ≥ 9 checklist items, while the remaining two studies [29, 32] met 7/10 items. The items with a lower number of “yes” were the item #6 (“Has the relationship between researcher and participants been adequately considered?”) that obtained 2 yes, 5 can’t tell, 1 not applicable, and the item #4 (“Was the recruitment strategy appropriate to the aims of the research?”) with 5 yes and 3 no.

Research question 3 (adherence)

The methodological quality of the study by Landfeldt et al. of 2015 [11] was assessed using the CASP Qualitative Studies Checklist tool [17]. The study fulfilled 9/10 items, while the item concerning the relationship between researcher and participants was deemed not applicable as the survey was managed without face-to-face interaction.

Research question 4 (economic impact)

The methodological quality of the included economic studies was assessed using the Consensus Health Economic Criteria (CHEC) tool [18] that includes 19 items formulated as questions that require a "yes/no" answer. For all three included studies, it was considered that 7/19 (37%) items were not applicable as they refer to cost-effectiveness studies. All three studies fulfilled the majority of the items, having negative responses only in one or two items. The study by Cavazza et al. [29] had positive answers to 10/12 (83%) applicable items; the two items not fulfilled were related to the lack of sensitivity analyses and the discussion on the generalizability of the results not clearly reported. Landfeldt et al. (2014) [31] received a positive answer to all the items deemed applicable, while for the study by Landfeldt et al. (2017) [37] 11/12 (92%) items obtained a positive answer and only 1/19 (8%) item got a negative answer due to lack of sensitivity analyses.
