# Supplementary material for: Duchenne muscular dystrophy in Italy: A systematic review of epidemiology, quality of life, treatment adherence, and economic impact
Source: PLoS One. 2023 Jun 27;18(6):e0287774. doi: 10.1371/journal.pone.0287774 (PMC10298760; doi:10.1371/journal.pone.0287774)
Supplement: Attachment — Submitted filename: Rebuttal letter.docx [file pone.0287774.s005.docx]

**Rebuttal letter**

Dear Editor,

Please find below our response to the motivation described in the decision letter to reject the manuscript PONE-D-22-28344 for publication in Plos One.

PONE-D-22-28344

Duchenne muscular dystrophy in Italy: a systematic review of epidemiology, quality of life, treatment adherence, and economic impact

PLOS ONE

Dear Dr. Orso,

Thank you for submitting your manuscript for review by PLOS ONE. After careful consideration, we have decided that your manuscript does not currently meet our standards for systematic and scoping reviews. Therefore, we are closing the file on your submission and rejecting your manuscript.

While PLOS ONE does consider systematic reviews and meta-analyses for publication, we require that these submissions address a clear and defined research question, conduct a systematic and comprehensive literature review; and use clearly reported, reproducible, and systematic methods to identify, select, and critically appraise relevant research. Our author guidelines for systematic reviews are at https://journals.plos.org/plosone/s/submission-guidelines#loc-systematic-reviews-and-meta-analyses.

In the case of your submission, we note that you applied the PRISMA checklist to assess the quality of the included systematic reviews. Please note that we do not consider the PRISMA checklist to be a quality assessment tool but rather a reporting checklist. Conclusions in a systematic review or meta-analysis should be related to the quality of the included publications. Without this critical assessment of the included studies, we do not feel that your systematic review currently fulfils our requirements for consideration (for more information, we would refer you to the following source: Higgins JPT, Thomas J, Chandler J, Cumpston M, Li T, Page MJ, Welch VA (editors). Cochrane Handbook for Systematic Reviews of Interventions version 6.2 (updated February 2021). Cochrane, 2021. Available from [www.training.cochrane.org/handbook](http://www.training.cochrane.org/handbook).

Authors response: We agreed with your comment and therefore we re-assessed the quality of the two systematic reviews included for the Research Question 1 (epidemiology) using the JBI Critical Appraisal Checklist for Systematic Reviews and Research Synthesis (<https://jbi.global/critical-appraisal-tools>). JBI critical appraisal checklists are widely used in the literature. The JBI checklist for systematic reviews [1] has 1,137 citations on Google Scholar (<https://scholar.google.com/scholar?hl=it&as_sdt=0%2C5&q=Summarizing+systematic+reviews%3A+methodological+development%2C+conduct+and+reporting+of+an+umbrella+review+approach&btnG>=).

As a result of these concerns, we cannot consider the manuscript for publication at PLOS ONE at this time. However, if you are able to address the concerns discussed above, we may be able to consider a revised version of the manuscript. This would however require a substantial amount of work and thus, would be considered as a new submission. If you are able to revise the manuscript to respond to the concerns noted above, please refer to the original submission in the covering letter and accompany the submission by a rebuttal letter that responds to the comments made on the original manuscript. In case of resubmitting a revised version of your manuscript, please ensure that all studies have been assessed by an appropriate quality assessment tool.

I am sorry that we cannot be more positive on this occasion, but I hope you appreciate the reasons for this decision.

Kind regards,

Johannes Stortz

Staff Editor

PLOS ONE

**Reference**

1.	Aromataris E, Fernandez R, Godfrey CM, Holly C, Khalil H, Tungpunkom P. Summarizing systematic reviews: methodological development, conduct and reporting of an umbrella review approach. Int J Evid Based Healthc. 2015;13(3):132-40.
