# Supplementary material for: Duchenne muscular dystrophy in Italy: A systematic review of epidemiology, quality of life, treatment adherence, and economic impact
Source: PLoS One. 2023 Jun 27;18(6):e0287774. doi: 10.1371/journal.pone.0287774 (PMC10298760; doi:10.1371/journal.pone.0287774)
Supplement: Attachment — Submitted filename: Response to Reviewers.docx [file pone.0287774.s006.docx]

**Response to Reviewers**

PONE-D-23-04901

Duchenne muscular dystrophy in Italy: a systematic review of epidemiology, quality of life, treatment adherence, and economic impact

PLOS ONE

Dear Dr. Massimiliano Orso,

Thank you for submitting your manuscript to PLOS ONE. After careful consideration, we feel that it has merit but does not fully meet PLOS ONE’s publication criteria as it currently stands. Therefore, we invite you to submit a revised version of the manuscript that addresses the points raised during the review process.

==============================

Please submit your revised manuscript by May 05 2023 11:59PM. If you will need more time than this to complete your revisions, please reply to this message or contact the journal office at plosone@plos.org. When you're ready to submit your revision, log on to https://www.editorialmanager.com/pone/ and select the 'Submissions Needing Revision' folder to locate your manuscript file.

Please include the following items when submitting your revised manuscript:

A rebuttal letter that responds to each point raised by the academic editor and reviewer(s). You should upload this letter as a separate file labeled 'Response to Reviewers'.

A marked-up copy of your manuscript that highlights changes made to the original version. You should upload this as a separate file labeled 'Revised Manuscript with Track Changes'.

An unmarked version of your revised paper without tracked changes. You should upload this as a separate file labeled 'Manuscript'.

If you would like to make changes to your financial disclosure, please include your updated statement in your cover letter. Guidelines for resubmitting your figure files are available below the reviewer comments at the end of this letter.

If applicable, we recommend that you deposit your laboratory protocols in protocols.io to enhance the reproducibility of your results. Protocols.io assigns your protocol its own identifier (DOI) so that it can be cited independently in the future. For instructions see: https://journals.plos.org/plosone/s/submission-guidelines#loc-laboratory-protocols. Additionally, PLOS ONE offers an option for publishing peer-reviewed Lab Protocol articles, which describe protocols hosted on protocols.io. Read more information on sharing protocols at https://plos.org/protocols?utm_medium=editorial-email&utm_source=authorletters&utm_campaign=protocols.

We look forward to receiving your revised manuscript.

Kind regards,

Omar Enzo Santangelo

Academic Editor

PLOS ONE

Journal Requirements:

When submitting your revision, we need you to address these additional requirements.

1. Please ensure that your manuscript meets PLOS ONE's style requirements, including those for file naming. The PLOS ONE style templates can be found at https://journals.plos.org/plosone/s/file?id=wjVg/PLOSOne_formatting_sample_main_body.pdf and

https://journals.plos.org/plosone/s/file?id=ba62/PLOSOne_formatting_sample_title_authors_affiliations.pdf

2. We note that you have included the phrase “data not shown” in your manuscript. Unfortunately, this does not meet our data sharing requirements. PLOS does not permit references to inaccessible data. We require that authors provide all relevant data within the paper, Supporting Information files, or in an acceptable, public repository. Please add a citation to support this phrase or upload the data that corresponds with these findings to a stable repository (such as Figshare or Dryad) and provide and URLs, DOIs, or accession numbers that may be used to access these data. Or, if the data are not a core part of the research being presented in your study, we ask that you remove the phrase that refers to these data.

RESPONSE: We deleted the sentence “data not shown” from the Table 2 because it was referred to the fact that in the study by Cavazza et al. EQ-5D reference values for the general population have not been reported. Authors only wrote: “*Therefore, the HRQOL of people with DMD is much lower than that of the general population*”, without reporting the actual EQ-5D scores for the general population.

[Note: HTML markup is below. Please do not edit.]

Reviewers' comments:

Reviewer's Responses to Questions

Comments to the Author

1. Is the manuscript technically sound, and do the data support the conclusions?

The manuscript must describe a technically sound piece of scientific research with data that supports the conclusions. Experiments must have been conducted rigorously, with appropriate controls, replication, and sample sizes. The conclusions must be drawn appropriately based on the data presented.

Reviewer #1: Yes

Reviewer #2: Yes

2. Has the statistical analysis been performed appropriately and rigorously?

Reviewer #1: N/A

Reviewer #2: N/A

3. Have the authors made all data underlying the findings in their manuscript fully available?

The PLOS Data policy requires authors to make all data underlying the findings described in their manuscript fully available without restriction, with rare exception (please refer to the Data Availability Statement in the manuscript PDF file). The data should be provided as part of the manuscript or its supporting information, or deposited to a public repository. For example, in addition to summary statistics, the data points behind means, medians and variance measures should be available. If there are restrictions on publicly sharing data—e.g. participant privacy or use of data from a third party—those must be specified.

Reviewer #1: Yes

Reviewer #2: Yes

4. Is the manuscript presented in an intelligible fashion and written in standard English?

PLOS ONE does not copyedit accepted manuscripts, so the language in submitted articles must be clear, correct, and unambiguous. Any typographical or grammatical errors should be corrected at revision, so please note any specific errors here.

Reviewer #1: Yes

Reviewer #2: No

5. Review Comments to the Author

Please use the space provided to explain your answers to the questions above. You may also include additional comments for the author, including concerns about dual publication, research ethics, or publication ethics. (Please upload your review as an attachment if it exceeds 20,000 characters)

Reviewer #1: Dear authors,

This paper was well-written and interesting. I think it will be a useful addition to the literature. The authors could not do a meta-analysis, but this is due to lack of literature. I do think the paper is too long. I see a potential to split it into two papers because there is sufficient material.

RESPONSE: Thank you for your positive comment. We agree that our paper is quite long and, in order to shorten the text, we decided to propose the quality assessment sections as supplementary material. This solution was accepted by the Editor. The paper is now substantially shorter and more readable.

We discussed with our co-authors the opportunity to split the paper in two and agreed to not do that as we prefer to publish a single paper in order to provide a comprehensive picture of DMD in Italy, describing the four areas together. We followed a similar approach in another review published on Plos One on paediatric growth hormone deficiency (GHD) in Italy (<https://journals.plos.org/plosone/article?id=10.1371/journal.pone.0264403>).

Kindly find my suggestions below:

Introduction

- In the introduction, the authors should clarify what their paper is expecting to add to the review and meta-analysis on the topic published by Crisafulli et al. I can see that this paper adds to the literature but it should be clearer for someone who doesn't know the previous literature. To me, the major contribution is the section on quality of life.

RESPONSE: Our systematic review was aimed to investigate the following four research questions: 1) the epidemiology of DMD in Italy; 2) the quality of life of patients with DMD and their caregivers in Italy; 3) the adherence to the different management strategies for DMD in Italy and to identify factors that could facilitate or hamper the adherence; 4) the economic impact of DMD in Italy.

As for the first research question (epidemiology), we included in our review both secondary studies (systematic or narrative reviews) and primary studies (cross-sectional, cohort) describing prevalence and/or incidence of DMD in Italy. Our aim was to summarize and update the existing evidence about epidemiology. Owing that among our included studies we found the recent systematic review and meta-analysis by Crisafulli et al., we described their results and in addition we described the results of another systematic review and those of an additional epidemiological primary study that met our inclusion criteria.

In conclusion, to address your comment, our review presents an evidence update, considering that Crisafulli et al. performed their searches in October 2019 and our search was performed in January 2023, and in addition includes updated evidence on the other research questions, particularly on the quality of life of DMD patients and their caregivers, as you pointed out. We slightly amended the text in different parts to reflect this.

Method

- Please replace "searches" with "a literature search" or similar throughout the paper.

RESPONSE: we amended the text as suggested.

- Please be more specific when you refer to adherence. Adherence to what? Pharmacotherapy? Physiotherapy? Occupational therapy? Arguably all are important for people with DMD.

RESPONSE: We were interested in assessing the adherence to all the management strategies for DMD, including those you mentioned. We have changed the text as follows: “3) *the adherence to the different management strategies for DMD in Italy, such as pharmacotherapy, physiotherapy and occupational therapy, and to identify factors that could facilitate or hamper the adherence*”.

- Overall the methods seem very well-conducted. Well done.

RESPONSE: Thank you for your positive comment.

Results

- I suggest the authors exercise caution in making a statement such as "In particular: for research question 1 (epidemiology), we found a systematic review with meta-analysis which was recently published and had good quality". If the authors assessed the quality of studies using GRADE or Newcastle-Ottawa/similar scales, these should be mentioned in the methods.

RESPONSE: We assessed the methodological quality of the systematic review authored by Crisafulli et al. using the JBI checklist, as explained later in the text (quality assessment sections; now included as supplementary material). We have included the following sentence in paragraph 3: “*In particular: for research question 1 (epidemiology), we found a recently published systematic review and meta-analysis that was judged to be of good quality, using the JBI Critical Appraisal Checklist for Systematic Reviews and Research Synthesis [15], and therefore we decided to present its results*”.

- Suggest being more specific on what the papers you identified about adherence concerned, as above. It is also necessary to be more specific when writing about adherence. For example, the authors write "The study highlighted a low adherence to the guidelines in all four countries." Which guidelines? Please be more specific.

RESPONSE: We added some information in the text, also citing the guidelines: “*To address the research question 3 (adherence), we identified only the study by Landfeldt et al. of 2015 [11], aimed at comparing the experience reported by the patient (or his family) regarding the medical management of DMD in Germany, Italy, UK and USA, with the recommendations of clinical guidelines* ***for the diagnosis and management of DMD, produced by an international, multidisciplinary group of experts [4, 36]****. A total of 770 patient-caregiver pairs completed the questionnaire; of these, 122 couples (16%) were Italian.* ***The questionnaire asked questions about the patient, his health status, visits to doctors and other healthcare providers related to DMD, clinical tests and assessments, drug use, and access to medical aids and devices***”.

Reviewer #2: This manuscript is a systematic review of studies addressing the epidemiology, quality of life, treatment adherence, and economic impact of Duchenne muscular dystrophy (DMD). While the authors do a reasonable job in reviewing the literature in the four broad areas, the manuscript is challenging to read. It is verbose, repetitive, and fails to summarize the information in a satisfactory manner.

RESPONSE: We agree that our paper is relatively long. However we think that this is due to the fact that we addressed four different research questions. In addition, we reported the results narratively, describing each study separately, and this had contributed to the manuscript length. A summary of results is reported in the Discussion section.

In order to shorten the text, in agreement with the Editor, we presented the quality assessment sections as supplementary material. In this way, the length of the paper has been significantly reduced.

The epidemiology portion seems problematic because the prevalence rates are estimated by different papers in different time windows. It is difficult to assess whether pooling these estimates makes sense.

RESPONSE: As for epidemiology, we included three studies: a systematic review and meta-analysis (Crisafulli et al.), another systematic review (Theadom et al.) and an observational epidemiological study (Mostacciuolo et al.). Crisafulli et al. reported pooled global DMD prevalence from 22 studies published between 1982 and 2016; they also reported pooled DMD birth prevalence from 29 studies published between 1977 and 2019. Heterogeneity was high in both meta-analyses (87% and 90%, respectively). Meta-regression was performed to investigate possible source of heterogeneity. The covariates selected were: the continent where the studies were carried out; the year the study began and its duration; the study design. As for the birth prevalence meta-analyses, the only covariate that significantly reduced the between-study heterogeneity (of about 45%) was the study period. In fact, older studies reported higher birth prevalence.

We added the following sentence in the text: “*All meta-analyses showed a substantial between-study heterogeneity (≥90%). Meta-regressions highlighted that the only covariate that reduced the heterogeneity of about 45% was the year in which the study began and its duration; older studies reported a higher DMD birth prevalence*”.

It might be helpful to have a professional editor rewrite the paper so that it is more effective at summarizing the retained studies. It might also be helpful to create a figure for each broad area to summarize the findings more visually.

RESPONSE: As explained in our previous response to your first comment, in agreement with the Editor, we presented the quality assessment sections as supplementary material. In this way, the length of the paper has been significantly reduced.

As for your suggestion to create a figure for each research question, we do not think that this is feasible; we had presented the PRISMA flow diagram showing the literature search process and we believe this help the readers in visualize the overall process (see Figure 1).

6. PLOS authors have the option to publish the peer review history of their article (what does this mean?). If published, this will include your full peer review and any attached files.

If you choose “no”, your identity will remain anonymous but your review may still be made public.

Do you want your identity to be public for this peer review? For information about this choice, including consent withdrawal, please see our Privacy Policy.

Reviewer #1: No

Reviewer #2: No

[NOTE: If reviewer comments were submitted as an attachment file, they will be attached to this email and accessible via the submission site. Please log into your account, locate the manuscript record, and check for the action link "View Attachments". If this link does not appear, there are no attachment files.]

While revising your submission, please upload your figure files to the Preflight Analysis and Conversion Engine (PACE) digital diagnostic tool, https://pacev2.apexcovantage.com/. PACE helps ensure that figures meet PLOS requirements. To use PACE, you must first register as a user. Registration is free. Then, login and navigate to the UPLOAD tab, where you will find detailed instructions on how to use the tool. If you encounter any issues or have any questions when using PACE, please email PLOS at figures@plos.org. Please note that Supporting Information files do not need this step.
